# Optimal State Transfer and Entanglement Generation in Power-Law Interacting Systems

**DOI:** 10.1103/physrevx.11.031016

**Published:** 2021-07

**Authors:** Minh C. Tran, Andrew Y. Guo, Abhinav Deshpande, Andrew Lucas, Alexey V. Gorshkov

**Affiliations:** 1 Joint Center for Quantum Information and Computer Science, NIST/University of Maryland, College Park, Maryland 20742, USA; 2 Joint Quantum Institute, NIST/University of Maryland, College Park, Maryland 20742, USA; 3 Department of Physics, University of Colorado, Boulder, Colorado 80309, USA; 4 Center for Theory of Quantum Matter, University of Colorado, Boulder, Colorado 80309, USA

**Keywords:** Atomic and Molecular Physics, Condensed Matter Physics, Quantum Information

## Abstract

We present an optimal protocol for encoding an unknown qubit state into a multiqubit Greenberger-Horne-Zeilinger-like state and, consequently, transferring quantum information in large systems exhibiting power-law $\left(1/r^{\alpha }\right)$ interactions. For all power-law exponents α between d and 2d+1, where d is the dimension of the system, the protocol yields a polynomial speed-up for α>2d and a superpolynomial speed-up for α≤2d, compared to the state of the art. For all α>d, the protocol saturates the Lieb-Robinson bounds (up to subpolynomial corrections), thereby establishing the optimality of the protocol and the tightness of the bounds in this regime. The protocol has a wide range of applications, including in quantum sensing, quantum computing, and preparation of topologically ordered states. In addition, the protocol provides a lower bound on the gate count in digital simulations of power-law interacting systems.

## I. INTRODUCTION

Harnessing entanglement between many particles is key to a quantum advantage in applications including sensing and timekeeping [1,2], secure communication [3], and quantum computing [4,5]. For example, encoding quantum information into a multiqubit Greenberger-Horne-Zeilinger-like (GHZ-like) state is particularly desirable as a subroutine in many quantum applications, including metrology [2], quantum computing [6,7], anonymous quantum communication [8,9], and quantum secret sharing [10].

The speed at which one can unitarily encode an unknown qubit state a|0⟩+b|1⟩ into a GHZ-like state a|00…0⟩+b|11…1⟩ of a large system is constrained by Lieb-Robinson bounds [11–25] and depends on the nature of the interactions in the system. In systems with finite-range interactions and power-law interactions decaying with distance r as $1/r^{\alpha }$ for all α≥2d+1, where d is the dimension of the system, the Lieb-Robinson bounds imply a linear light cone for the propagation of quantum information [23,25]. Consequently, in such systems, the linear size of a GHZ-like state that can be prepared from unentangled particles cannot grow faster than linearly with time.

The Lieb-Robinson bounds become less stringent for longer-range interactions, i.e., those with α<2d+1. The bounds theoretically allow quantum information to travel a distance r in time t that scales sublinearly with r [14–16,21,26]. However, no protocol in the present literature can saturate these bounds. In particular, existing protocols for α∈(d,2d] are exponentially slower than what is allowed by the corresponding bounds. Up until now, the existence of this gap between the Lieb-Robinson bounds and the achievable protocols has meant that at least one of the two is not yet optimal, hinting at either a tighter Lieb-Robinson bound or the possibility of speeding up many quantum information processing tasks.

In this paper, we close the gap for all α∈(d,2d+1) in d dimensions by designing a protocol for encoding an arbitrary qubit into a multiqubit GHZ-like state and, subsequently, transferring information at the limits imposed by the Lieb-Robinson bounds. There are three key implications of the protocol. First, within these regimes of α, it establishes the tightness of the Lieb-Robinson bounds, up to subpolynomial corrections, and effectively puts an end to the 15-year search for a tighter bound. Second, our protocol implies optimal designs for future experiments on power-law interacting systems, including trapped ions [27,28] (α∈[0,3]) in one and two dimensions [29], ultracold atoms in photonic crystals [32,33], van der Waals interacting Rydberg atoms [34,35] (α=6) in three dimensions [36], as well as the very common case of dipolar interactions in nitrogen-vacancy centers [37], polar molecules [38], and dipole-dipole interacting Rydberg atoms [39] (α=3) in two dimensions. Finally, our protocol implies a lower bound on the gate count in simulating power-law interacting systems on a quantum computer, providing a benchmark for the performance of quantum simulation algorithms.

The structure of the paper is as follows. In Sec. II, we define our setting and introduce the main result: the optimal state-transfer time in power-law interacting systems [Eq. (3)]. In Sec. III, we describe the corresponding optimal protocol for generating entanglement and subsequently transferring quantum information. At the end of Sec. III, we discuss the key ingredients that make the protocol outperform previously known protocols. Readers who are interested in the conceptual implications of the protocol may also skip ahead to Sec. IV, where we establish the tightness of existing Lieb-Robinson bounds and discuss implications for other types of speed limits associated with quantum information propagation.

## II. SETUP AND RESULTS

We first describe the setting of the problem and the main result in this section. For simplicity, we consider a d-dimensional hypercubic lattice Λ and a two-level system located at every site of the lattice. Our protocol generalizes straightforwardly to all regular lattices. Without loss of generality, we assume that the lattice spacing is one. We consider a power-law interacting Hamiltonian $H\left(t\right)=\sum_{i,j\in \Lambda }h_{ij}\left(t\right)$, where $h_{ij}\left(t\right)$ is a Hamiltonian supported on sites i,j such that, at all times t and for all i≠j, we have $\left\|h_{ij}\right\|\leq 1/\text{dist}{\left(i,j\right)}^{\alpha }$, where dist(i,j) is the distance between i,j,‖⋅‖ is the operator norm, and α≥0 is a constant. We use $|\text{GHZ}\left(a,b\right)\rangle_{S}$ to denote the GHZ-like state over sites in S⊆Λ:

(1) $$\left|\text{GHZ}\left(a,b\right)\rangle_{S}\equiv a\right|\bar{0}\rangle_{S}+b|\bar{1}\rangle_{S},$$

where $\left|\bar{x}\rangle_{S}\equiv {⊗}_{j\in S}\right|x\rangle_{j}\left(x=0,1\right)$ are product states over all sites in S and a,b are complex numbers such that $\left|a{|}^{2}+\right|b{|}^{2}=1$. In particular, we use |GHZ⟩ to denote the symmetric state $a=b=1/\sqrt{2}$.

Given a d-dimensional hypercube C⊆Λ of length r≥1, we consider the task of encoding a possibly unknown state a|0⟩+b|1⟩ of a site c∈C into the GHZ-like state $|\text{GHZ}\left(a,b\right)\rangle_{C}$ over C, assuming that all sites in C, except for c, are initially in the state |0⟩. Specifically, we construct a time-dependent, power-law interacting Hamiltonian H(t) that generates $U\left(t\right)=𝒯\text{ exp}\left[-i\int_{0}^{t}dsH\left(s\right)\right]$ satisfying

(2) $$U\left(t\right)(a\left|0\rangle +\rangle b\right|1\rangle {)}_{c}\left|\bar{0}\rangle_{C\setminus c}=a\right|\bar{0}\rangle_{C}+b|\bar{1}\rangle_{C}$$

at time

(3) $$t\left(r\right)\leq K_{\alpha }\times \{\begin{array}{ll} {\text{log}}^{K_{\alpha }r} & \text{ if }d<\alpha <2d\\ e^{\gamma \sqrt{\text{log }r}} & \text{ if }\alpha =2d\\ r^{\alpha -2d} & \text{ if }2d<\alpha \leq 2d+1. \end{array}$$

Here, $\gamma =3\sqrt{d},\kappa_{\alpha }$, and $K_{\alpha }$ are constants independent of t and r. Additionally, by reversing the unitary in Eq. (2) to “concentrate” the information in |GHZ(a,b)⟩ onto a different site in C, we can transfer a quantum state from c∈C to any other site $c^{\prime }\in C$ in time 2t.

## III. OPTIMAL PROTOCOL

**FIG. 1.**
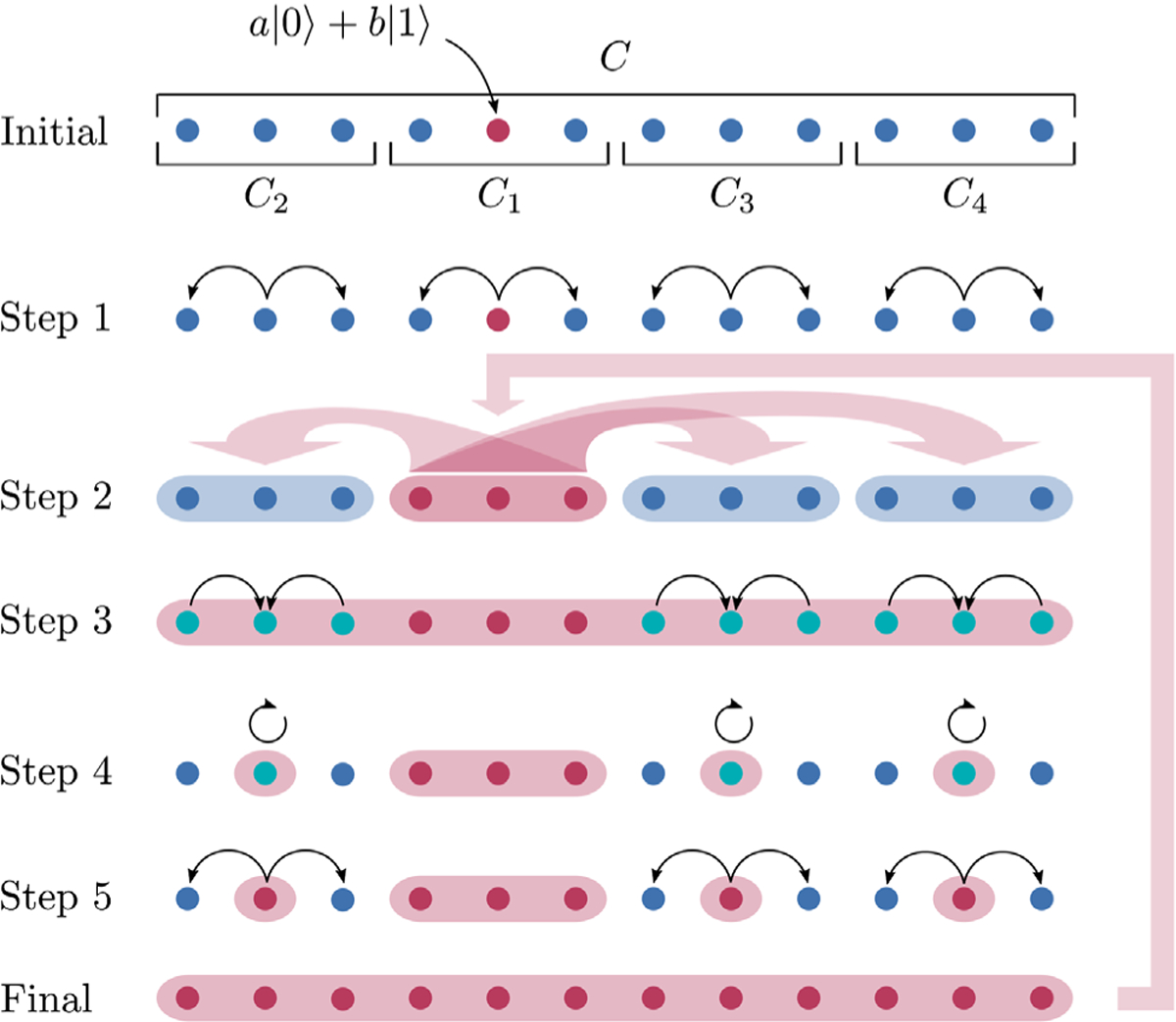
A demonstration of our protocol for encoding a qubit into a GHZ-like state in a one-dimensional system C. Initially, the unknown coefficients a,b are encoded in one qubit (red circle) while the other qubits are each initialized in state |0⟩. The first step of the protocol assumes the ability to encode information into GHZ-like states in subsystems $C_{1},\ldots ,C_{4}$ using, for example, nearest-neighbor interactions. In step 2, we apply a generalized controlled-phase gate [Eq. (6)] between the subsystems to “merge” the GHZ-like states into an entangled state between all sites. The last three steps rotate this entangled state into the desired GHZ-like state by concentrating the entanglement in each subsystem onto one qubit, applying single-qubit rotations, and redistributing the entanglement to the rest of the system. Repeatedly feeding the resulting GHZ-like state back into step 2 of the protocol yields larger and larger GHZ-like states.

The key idea of our protocol (Fig. 1) is to recursively build the GHZ-like state in a large hypercube from the GHZ-like states of smaller hypercubes. For the base case, we note that hypercubes of finite lengths, i.e., $r\leq r_{0}$ for some fixed $r_{0}$, can always be generated in times that satisfy Eq. (3) for some suitably large (but constant) prefactor $K_{\alpha }$. Assuming that we can encode information into a GHZ-like state in hypercubes of length $r_{1}$ in time $t_{1}$ satisfying Eq. (3), the following subroutine encodes information into a GHZ-like state in an arbitrary hypercube C of length $r=mr_{1}$ containing c—the site initially holding the phase information a,b. Here m is an α-dependent number to be chosen later.

*Step 1*.—We divide the hypercube C into $m^{d}$ smaller hypercubes $C_{1},\ldots ,C_{m^{d}}$, each of length $r_{1}$. Without loss of generality, let $C_{1}$ be the hypercube that contains c. Let $V=r_{1}^{d}$ be the number of sites in each $C_{j}$. In this step, we simultaneously encode a,b into $|\text{GHZ}\left(a,b\right)\rangle_{C_{1}}$ and prepare $|\text{GHZ}\rangle_{C_{j}}$ for all $j=2,\ldots ,m^{d}$, which, by our assumption, takes time:

(4) $$t_{1}\leq K_{\alpha }\times \{\begin{array}{ll} {\text{log}}^{K_{\alpha }}r_{1} & \text{ if }d<\alpha <2d\\ e^{\gamma \sqrt{\text{log }r_{1}}} & \text{ if }\alpha =2d\\ r_{1}^{\alpha -2d} & \text{ if }2d<\alpha \leq 2d+1. \end{array}$$

By the end of this step, the hypercube C is in the state

(5) $$(a\left|\bar{0}\rangle +b\right|\bar{1}\rangle {)}_{C_{1}}\overset{m_{d}}{\underset{j=2}{⊗}}\frac{\left|\bar{0}\rangle_{C_{j}}+\right|\bar{1}\rangle_{C_{j}}}{\sqrt{2}}.$$

*Step 2*.—Next, we apply the following Hamiltonian to the hypercube C:

(6) $$H_{2}=\frac{1}{{\left(mr_{1}\sqrt{d}\right)}^{\alpha }}\overset{m^{d}}{\underset{j=2}{\sum }}\underset{\mu \in C_{1}}{\sum }\underset{\nu \in C_{j}}{\sum }|1\rangle {\langle 1|}_{\mu }⊗|1{\rangle \langle 1|}_{\nu }.$$

This Hamiltonian effectively generates the so-called controlled-phase gate between the hypercubes, with $C_{1}$ being the control hypercube and $C_{2},\ldots ,C_{m^{d}}$ being the target hypercubes. We choose the interactions between qubits in Eq. (6) to be identical for simplicity. If the interactions were to vary between qubits, we would simply turn off the interaction between $C_{1}$ and $C_{j}$ once the total phase accumulated by $C_{j}$ reaches π [40]. The prefactor $1/{\left(mr_{1}\sqrt{d}\right)}^{\alpha }$ ensures that this Hamiltonian satisfies the condition of a power-law interacting Hamiltonian. It is straightforward to verify that, under this evolution, the state of the hypercube C rotates to

(7) $$a\left|\bar{0}\rangle_{C_{1}}\overset{m_{d}}{\underset{j=2}{⊗}}\frac{\left|\bar{0}\rangle_{C_{j}}+\right|\bar{1}\rangle_{C_{j}}}{\sqrt{2}}+b\right|\bar{1}\rangle_{C_{1}}\overset{m_{d}}{\underset{j=2}{⊗}}\frac{\left|\bar{0}\rangle_{C_{j}}-\right|\bar{1}\rangle_{C_{j}}}{\sqrt{2}}$$

after time $t_{2}=\pi d^{\alpha /2}{\left(mr_{1}\right)}^{\alpha }/V^{2}$.

The role of power-law interactions in our protocol can be inferred from the value of $t_{2}$. Intuitively, the speed of simultaneously entangling hypercube $C_{1}$ with hypercubes $C_{2},\ldots ,C_{m^{d}}$ is enhanced by the $V^{2}=r_{1}^{2d}$ couplings between the hypercubes. However, the strength of each coupling, proportional to $1/{\left(mr_{1}\right)}^{\alpha }$, is suppressed by the maximum distance between the sites in $C_{1}$ and those in $C_{2},\ldots ,C_{m^{d}}$. With a small enough α, the enhancement due to $V^{2}$ overcomes the suppression of power-law interactions, resulting in a small entanglement time $t_{2}$. In particular, when $\alpha <2d,t_{2}$ actually *decreases* with $r_{1}$, implying that step 2 would be faster in later iterations if we were to keep m constant.

To obtain the desired state $|\text{GHZ}\left(a,b\right)\rangle_{C}$, it remains to apply a Hadamard gate on the effective qubit $\left\{\left|\bar{0}\rangle_{C_{j}},\right|\bar{1}\rangle_{C_{j}}\right\}$ for $j=2,\ldots ,m^{d}$. We do this in the following three steps by first concentrating the information stored in hypercube $C_{j}$ onto a single site $c_{j}\in C_{j}$ (step 3), then applying a Hadamard gate on $c_{j}$ (step 4), and then unfolding the information back onto the full hypercube $C_{j}$ (step 5).

*Step 3*.—By our assumption, for each hypercube $C_{j}\;\left(j=2,\ldots ,m^{d}\right)$ and given a designated site $c_{j}\in C_{j}$, there exists a (time-dependent) Hamiltonian $H_{j}$ that generates a unitary $U_{j}$ such that

(8) $${\left(\psi_{0}\left|0\rangle +\psi_{1}\right|1\rangle \right)}_{c_{j}}\left|\bar{0}\rangle_{C_{j}\setminus c_{j}}\overset{U_{j}}{\to }\psi_{0}\right|\bar{0}\rangle_{C_{j}}+\psi_{1}|\bar{1}\rangle_{C_{j}},$$

for all complex coefficients $\psi_{0}$ and $\psi_{1}$, in time $t_{1}$ satisfying Eq. (4). By linearity, this property applies even if $C_{j}$ is entangled with other hypercubes. Consequently, backward time evolution under $H_{j}$ generates $U_{j}^{†}$, which “undoes” the GHZ-like state of the jth hypercube:

(9) $$\psi_{0}\left|\bar{0}\rangle_{C_{j}}+\psi_{1}\right|\bar{1}\rangle_{C_{j}}\overset{U_{j}^{†}}{\to }{\left(\psi_{0}\left|0\rangle +\psi_{1}\right|1\rangle \right)}_{c_{j}}|\bar{0}\rangle_{C_{j}\setminus c_{j}},$$

for any $\psi_{0},\psi_{1}$. In this step, we simultaneously apply $U_{j}^{†}$ to $C_{j}$ for all $j=2,\ldots ,m^{d}$. These unitaries rotate the state of C to

(10) $$a\left|\bar{0}\rangle_{C_{1}}\overset{m^{d}}{\underset{j=2}{⊗}}\left|+\rangle_{c_{j}}\right|\bar{0}\rangle_{C_{j}\setminus c_{j}}+b\right|\bar{1}\rangle_{C_{1}}\overset{m^{d}}{\underset{j=2}{⊗}}\left|-\rangle_{c_{j}}\right|\bar{0}\rangle_{C_{j}\setminus c_{j}},$$

where $|\pm \rangle =\left(\left|0\rangle \pm \right|1\rangle \right)/\sqrt{2}$.

*Step 4*.—We then apply a Hadamard gate, i.e.,

(11) $$\frac{1}{\sqrt{2}}\left(\begin{array}{cc} 1 & 1\\ 1 & -1 \end{array}\right),$$

to the site $c_{j}$ of each hypercube $C_{j},j=2,\ldots ,m^{d}$. These Hadamard gates can be implemented arbitrarily fast since we do not assume any constraints on the single-site terms of the Hamiltonian. The state of C by the end of this step is

(12) $$a\left|\bar{0}\rangle_{C_{1}}\overset{m^{d}}{\underset{j=2}{⊗}}\left|0\rangle_{c_{j}}\right|\bar{0}\rangle_{C_{j}\setminus c_{j}}+b\right|\bar{1}\rangle_{C_{1}}\overset{m^{d}}{\underset{j=2}{⊗}}\left|1\rangle_{c_{j}}\right|\bar{0}\rangle_{C_{j}\setminus c_{j}}.$$

*Step 5*.—Finally, we apply $U_{j}$ again to each hypercube $C_{j}\left(j=2,\ldots ,m^{d}\right)$ to obtain the desired GHZ-like state:

(13) $$a|\bar{0}\rangle_{C_{1}}\overset{m^{d}}{\underset{j=2}{⊗}}\left|\bar{0}\rangle_{C_{j}}+b\right|\bar{1}\rangle_{C_{1}}\overset{m^{d}}{\underset{j=2}{⊗}}\left|\bar{1}\rangle_{C_{j}}=\right|\text{GHZ}\left(a,b\right)\rangle_{C}.$$

At the end of this routine, we have implemented the unitary satisfying Eq. (2) in time:

(14) $$t=3t_{1}+t_{2}=3t_{1}+\pi d^{\alpha /2}m^{\alpha }r_{1}^{\alpha -2d}.$$

We now consider three cases corresponding to different ranges of α and show that if $t_{1}\left(r_{1}\right)$ satisfies Eq. (3), then t(r) also satisfies Eq. (3).

For α∈(2d,2d+1], we have $t_{1}\leq K_{\alpha }r_{1}^{\alpha -2d}$. Choosing m>1 to be a constant integer, we have

(15) $$t\leq \left(\frac{3K_{\alpha }}{m^{\alpha -2d}}+\pi d^{\alpha /2}m^{2d}\right){\left(mr_{1}\right)}^{\alpha -2d}\leq K_{\alpha }r^{\alpha -2d},$$

where we require $m>3^{1/\left(\alpha -2d\right)}$ and choose

(16) $$K_{\alpha }\geq \frac{\pi d^{\alpha /2}m^{2d}}{1-\frac{3}{m^{\alpha -2d}}}=\frac{\pi d^{\alpha /2}m^{\alpha }}{m^{\alpha -2d}-3}.$$

For α∈(d,2d), we choose m to scale with $r_{1}$ such that $r_{1}^{\lambda -1}<m\leq 2r_{1}^{\lambda -1}$, where λ=2d/α. The length of the larger cube C is then $r=mr_{1}>r_{1}^{\lambda }$ and, therefore, the total time is

(17) $$t\leq 3K_{\alpha }{\text{log}}^{\kappa_{\alpha }}\;r_{1}+\pi {\left(2\sqrt{d}\right)}^{\alpha }r_{1}^{\left(\lambda -1\right)\alpha +\alpha -2d}$$

(18) $$\leq \frac{4K_{\alpha }}{\lambda^{\kappa_{\alpha }}}{\text{log}}^{\kappa_{\alpha }}\left(r_{1}^{\lambda }\right)\leq K_{\alpha }{\text{log}}^{\kappa_{\alpha }}r,$$

where we choose $\kappa_{\alpha }=\text{log }4/\text{log}\left(2d/\alpha \right)$ and assume $K_{\alpha }{\text{log}}^{\kappa_{\alpha }}r_{1}\geq \pi {\left(2\sqrt{d}\right)}^{\alpha }$ to simplify the expression. We note that the factor log 4 in the definition of $\kappa_{\alpha }$ can be made arbitrarily close to log 3 by increasing $K_{\alpha }$.

Finally, for α=2d, we choose m such that $\text{exp}\left[\left(\gamma /2d\right)\sqrt{\text{log }r_{1}}\right]\leq m\leq 2\text{ exp}\left[\left(\gamma /2d\right)\sqrt{\text{log }r_{1}}\right]$, where $\gamma =3\sqrt{d}$. Substituting $t_{1}\leq K_{\alpha }\text{exp}\left(\gamma \sqrt{\text{log }r_{1}}\right)$ into Eq. (14), we have

(19) $$t\leq \left(3K_{\alpha }+2^{\alpha }\pi d^{\alpha /2}\right)e^{\gamma \sqrt{\text{log }r_{1}}}.$$

Assuming $r_{1}\geq \text{exp}\left(8/d\right)$, it is straightforward to prove that $\gamma \sqrt{\text{log }r_{1}}\leq \gamma \sqrt{\text{log}\left(mr_{1}\right)}-2$. Applying this condition on the above inequality, we have

(20) $$t\leq \frac{1}{e^{2}}\left(3K_{\alpha }+2^{\alpha }\pi d^{\alpha /2}\right)e^{\gamma \sqrt{\text{log }r}}\leq K_{\alpha }e^{\gamma \sqrt{\text{log }r}},$$

where $r=mr_{1}$ is the length of the resulting GHZ-like state and we chose $K_{\alpha }\geq 2^{\alpha }\pi d^{\alpha /2}/\left(e^{2}-3\right)$. Equations (15), (18), and (20) prove that t satisfies Eq. (3). Repeatedly applying this routine yields larger and larger GHZ-like states.

Before discussing the implications of our protocol, we would like to explain intuitively the main sources of its improvement relative to existing protocols. In our protocol, we simultaneously encode the information into the GHZ-like state over $C_{1}$ and create the symmetric GHZ states over other multiqubit subsystems $C_{2},\ldots ,C_{m^{d}}$. As a result, the implementation of the controlled operations in step 2 (Fig. 1) is enhanced quadratically by the volume of each subsystem. In contrast, the protocol in Ref. [41] applies controlled operations between a large subsystem and individual remaining sites of the system, resulting in the implementation time scaling only linearly with the volume of the subsystem.

On the other hand, while the state transfer protocol in Refs. [24,25] also applies controlled operations between large subsystems and is, therefore, sped up quadratically by the subsystem volume, it only uses qubits in small neighborhoods around the source and the target of the transfer. In our protocol, we maximize the size of the resulting GHZ-like state at the end of each iteration by allowing m to depend on α and on the size of the existing GHZ-like states. When we use the protocol for state transfer, this strategy results in most of the qubits between the source and the target sites participating in the transfer, significantly speeding up the protocol.

## IV. DISCUSSION

We now discuss the performance and the implications of our protocol (summarized in Table I). First, our protocol allows for encoding an unknown qubit into a multiqubit GHZ-like state and, subsequently, performing state transfer at unprecedented speeds. For d<α<2d, which applies, for example, to dipole-dipole interactions (α=3) in two dimensions and to the effective interactions between trapped ions (α∈[0,3]) in one and two dimensions, our protocol encodes information into GHZ-like states and transfers information in polylogarithmic time, exponentially faster than protocols available in the literature. Even for the seemingly weakly long-range interactions with α=2d, such as van der Waals interactions between Rydberg atoms (α=6) in three dimensions, our protocol still takes only subpolynomial time to entangle an entire system and to transfer a quantum state. When applied to the preparation of GHZ states, these speed-ups enable potential improvements to quantum sensors built from nitrogen-vacancy centers [42,43], Rydberg atoms [44,45], and polar molecules [46], as well as to atomic clocks based on trapped ions [47].

### Optimal quantum information processing.—

The optimality of our protocol for α∈(d,2d+1) in d dimensions also lays the foundation for optimal quantum information processing in power-law interacting systems [48,49]. Using quantum state transfer between auxiliary qubits and encoding qubits into large GHZ-like states as subroutines, our protocol leads to optimal implementations of quantum gates between distant qubits in large quantum computers. In particular, the faster encoding of information into a GHZ-like state of ancillary qubits speeds up [7] the implementation of the quantum fan-out—a powerful multiqubit quantum gate [50]. At the same time, the faster state transfer speeds up [41] the constructions of multiscale entanglement renormalization ansatz (MERA) states, commonly used to represent highly entangled—including topologically ordered [51]—states [52–54]. Specifically, we can implement a fan-out gate [7] on qubits in a hypercube of volume n and prepare a MERA state [41] on these qubits in time t~polylog (n) for $\alpha \in \left(d,2d\right),t\sim e^{\left(\gamma /\sqrt{d}\right)\sqrt{\text{log }n}}$ for α=2d—which are both exponential speed-ups compared to the previous best—and $t\sim n^{\left(\alpha -2d\right)/d}$ for α∈(2d,2d+1). The optimality of these operations is again guaranteed (up to subpolynomial corrections) by the matching lower limits imposed by the Lieb-Robinson bounds [7,41].

In practice, using single-site Hamiltonians to implement the echoing technique of Ref. [41], the controlled-phase gate in step 2 of our protocol can be realized starting from time-independent power-law interactions between all sites of the system. The protocol therefore does not require explicit time-dependent control of individual two-qubit Hamiltonians, making it appealing for implementation on available experimental platforms. However, because the diameter of the GHZ-like state increases by more than twofold in every iteration of the protocol, the scaling in Eq. (3) may only be observed in large systems.

### Information-propagation speed limits.—

Conceptually, since our protocol saturates (up to subpolynomial corrections) the Lieb-Robinson bounds for d<α<2d+1 for all d, we demonstrate, for the first time, the tightness of these fundamental bounds in these regimes. In particular, the subpolynomial entanglement time for α≤2d disproves the conjecture in Refs. [55,56], where a gap in the understanding of the heating times and the effective generators of dynamics in periodically driven, power-law interacting systems had suggested the existence of a tighter Lieb-Robinson bound with an algebraic light cone in this regime of α. We discuss in more detail below what could have resulted in this gap in our understanding.

Since the best-known generalizations of these bounds to k-body, power-law interacting Hamiltonians—those described by $H=\sum_{X}h_{X}$, where the sum is over all subsets X⊂Λ of at most k sites and $\sum_{X∋i,j}\left\|h_{X}\right\|\leq 1/\text{dist}{\left(i,j\right)}^{\alpha }$ for all i≠j—have the same scaling as the best-known two-body bounds when d<α<2d+1 [14] (see also Table I), the scaling of our two-body protocol is also optimal even if one allows for k-body interactions. In other words, in this regime of α, allowing for k-body interactions cannot enable a qualitative speed-up relative to two-body interactions.

Our protocol also generalizes straightforwardly from two-level to arbitrary finite-level systems. Given a q-level system at each site of the lattice, we can unitarily encode an arbitrary state $\left|\psi \rangle_{c}=\sum_{\ell =0}^{q-1}a_{\ell }\right|\ell \rangle$ of site c∈C, where $a_{\ell }$ are complex coefficients and C is a hypercube of linear size r, into a multiqudit state,

(21) $$\left|\psi \rangle_{c}\right|\bar{0}\rangle_{C\setminus c}\to \overset{q-1}{\underset{\ell =0}{\sum }}a_{\ell }|\bar{\ell }\rangle_{C},$$

in time t(r) satisfying Eq. (3). This can be done by replacing the Hamiltonian in Eq. (6) with

(22) $$\frac{1}{{\left(mr_{1}\sqrt{d}\right)}^{\alpha }}\overset{m^{d}}{\underset{j=2}{\sum }}\underset{\mu \in C_{1}}{\sum }\underset{\nu \in C_{j}}{\sum }\overset{q-1}{\underset{\ell ,\ell^{\prime }=0}{\sum }}\ell \ell^{\prime }|\ell \rangle {\langle \ell |}_{\mu }⊗|\ell^{\prime }\rangle {\langle \ell^{\prime }|}_{\nu }$$

and replacing the single-qubit Hadamard gate in step 4 by a q-by-q discrete Fourier transform matrix. Since the Lieb-Robinson bounds have the same light cones for any finite-level systems, our protocol also saturates these bounds for α∈(d,2d+1) in d dimensions.

In our protocol, we assume that a|0⟩+b|1⟩ is a possibly unknown state. Encoding such a state into the GHZ-like state is at least as hard as generating a GHZ-like state with known coefficients a,b. In fact, the latter task is not known to be sufficient for state transfer and, therefore, is not *directly* constrained by the Lieb-Robinson bounds. Instead, one often indirectly obtains a speed limit for this task by applying the Lieb-Robinson bounds on the growth of two-point connected correlators [14,24,57]. Our protocol for encoding into a GHZ-like state saturates (up to subpolynomial corrections) the bounds on the growth of connected correlators [14,26,57] when d<α≤2d+1 (see Table I), confirming that knowing the coefficients a,b does not speed up the preparation of the GHZ-like state in this regime.

We also note that our protocol violates the so-called Frobenius light cone, initially derived in Ref. [24] for α>3/2 in one dimension as part of a hierarchy of speed limits for different types of information propagation in long-range interacting systems and later extended to regimes of smaller α in Ref. [58,59]. The Frobenius bound, which considers information propagation from the operator-spreading perspective, constrains information-propagation tasks that are more demanding than the tasks that saturate the Lieb-Robinson bound, and therefore has a more stringent light cone. For example, quantum state transfer given intermediate qubits in arbitrary initial states (i.e., universal state transfer) is constrained by the Frobenius light cone, whereas state transfer assuming initialized intermediate qubits is constrained by the Lieb-Robinson bound and can actually violate the Frobenius light cone [24] (see also Table I). Determining which of the bounds tightly constrains a given task is still an active area of research. The protocol in this paper proves for the first time that the task of encoding information into GHZ-like state—which is at least as hard as state transfer with initialization—is not constrained by the Frobenius light cone, but is instead tightly constrained (up to subpolynomial corrections) by the Lieb-Robinson bound. In particular, when d<α<2d, our protocol proves that state transfer with initialization can be implemented exponentially faster than state transfer without initialization, which is constrained by polynomial light cones in this regime [24,58]. Furthermore, since our protocol for encoding into a GHZ-like state can also be used to prepare a known GHZ-like state, our protocol also proves for the first time that preparing a known GHZ-like state is not constrained by the Frobenius light cone.

An important open question is whether there exists a *time-independent* power-law Hamiltonian that propagates information at the same speed as our protocol does. Such a Hamiltonian would enable observation of fast information propagation in existing experimental platforms where arbitrary time-dependent control is often not available. On the other hand, the lack of such a Hamiltonian would imply a more stringent speed limit for time-independent Hamiltonians than the one given by the Lieb-Robinson bound. Such a speed limit may in turn imply that the effective time-independent Floquet Hamiltonians constructed in Refs. [55,56,60] for periodically driven, power-law interacting systems would correctly generate the dynamics of local observables even in the regime d<α<2d, closing the aforementioned gap in our understanding. These observations emphasize the need for studies, in a similar spirit to Ref. [24], of fundamental speed limits for time-independent Hamiltonians and, more generally, systems under various physical constraints.

Another interesting open question is whether our optimal protocol can be generalized to the regime 0≤α≤d, where there are still substantial gaps between the Lieb-Robinson bounds and achievable protocols [17,41,61–63]. The bounds suggest that, in addition to the distance, the information-propagation time also depends on the total number of sites on the lattice. Consequently, we would expect an optimal protocol to make use of all sites on the lattice, including those that are far from both the source and the target of the propagation. We consider such a generalization an important future direction.

### Resource lower bound for quantum simulation.—

Our protocol also gives the first known example of a lower bound on the gate count for simulating power-law systems on a quantum computer: it takes Ω(n) elementary quantum gates to simulate an n-qubit power-law system evolving for time $t\geq t_{*}$, where

(23) $$t_{*}=\{\begin{array}{ll} \Theta \left({\text{log}}^{k_{\alpha }}n\right) & \text{ if }d<\alpha <2d\\ \Theta \left(e^{\gamma \sqrt{\left(\text{log }n\right)/d}}\right) & \text{ if }\alpha =2d\\ \Theta \left(n^{\alpha /d-2}\right) & \text{ if }2d<\alpha \leq 2d+1, \end{array}$$

to constant error. Indeed, if an algorithm could use fewer than Ω(n) quantum gates to perform the simulation for times within $t=t_{*}$ satisfying Eq. (23), we could use the algorithm to simulate our protocol and prepare an n-qubit GHZ state. However, since an n-qubit GHZ state must take Ω(n) quantum gates to prepare, we would arrive at a contradiction.

Lower bounds on the simulation gate count are valuable benchmarks for the performance of quantum algorithms. Reference [64] gives an algorithm for simulating the time evolution of finite-range interacting Hamiltonians, the gate count of which was shown to be optimal via a matching lower bound. To date, despite progressively more efficient quantum simulation algorithms [21,65] in recent literature, no saturable lower bounds are known for power-law systems. For example, the analysis of the Suzuki-Trotter product formulas in Ref. [65] results in upper bounds,

(24) $$g_{\alpha }=\{\begin{array}{ll} O\left(n^{2+o\left(1\right)}t^{1+o\left(1\right)}\right) & \text{ if }d<\alpha \leq 2d\\ O\left({\left(nt\right)}^{1+d/\left(\alpha -d\right)+o\left(1\right)}\right) & \text{ if }\alpha >2d, \end{array}$$

for simulating an n-qubit power-law system for time t. At $t=t_{*}$ given in Eq. (23), the corresponding upper bounds reduce to

(25) $$g_{\alpha }=\{\begin{array}{ll} O\left(n^{2+o\left(1\right)}\right) & \text{ if }d<\alpha \leq 2d\\ O\left(n^{\alpha /d+o\left(1\right)}\right) & \text{ if }2d<\alpha \leq 2d+1. \end{array}$$

The gap between this state-of-the-art upper bound and our lower bound Ω(n) hints at the possibility of a more efficient algorithm for simulating power-law systems.

## Figures and Tables

**TABLE I.** A summary of known bounds and protocols in the regime α∈(d,2d+1) for several information-propagation tasks: encoding an unknown qubit state into a GHZ-like state (row 1), preparing a known GHZ-like state (row 2), state transfer assuming we can initialize intermediate qubits (row 3), and state transfer given intermediate qubits in arbitrary states (i.e., so-called universal state transfer [24], row 4). The tasks of encoding information into GHZ-like states, preparing a known GHZ-like state, and quantum state transfer with initialization are constrained by the Lieb-Robinson bounds. On the other hand, state transfer given intermediate qubits in arbitrary states (i.e., universal state transfer) is more difficult than state transfer with initialized intermediate qubits and is bounded by the more stringent Frobenius light cone [24]. The bounds on encoding information into GHZ-like states (except Ref. [23]) also apply to general k-body interactions. All listed bounds also hold not just for qubits, but for all finite-level systems. For d<α<2d+1, our protocol saturates (up to subpolynomial corrections) the known bounds, thus proving the optimality of both the protocol and the bounds.

Tasks	Known light cones	Previous best protocols	Our protocol
Encoding into a GHZ-like state	$t≳\{\begin{array}{ll} \text{log }r & \alpha \in (d,2d]\left[14\right]\\ r^{\alpha -2d} & \alpha \in \left(2d,2d+1\right)\left[23,26\right] \end{array}$	$t≳\{\begin{array}{ll} r^{\alpha -d} & \alpha \in \left(d+1\right)\left[41\right]\\ r & \alpha \in [d+1,2d+1) \end{array}$	$t\sim \{\begin{array}{ll} \text{polylog }(r) & \alpha \in (d,2d)\\ e^{\gamma \sqrt{\text{log }r}} & \alpha =2d\\ r^{\alpha -2d} & \alpha \in (2d,2d+1) \end{array}$
Preparing a known GHZ-like state	$t≳\{\begin{array}{ll} \text{log }r & \alpha \in (d,2d]\left[14,57\right]\\ r^{\alpha -2d} & \alpha \in \left(2d,2d+1\right)\left[26,57\right] \end{array}$	Same as encoding into a GHZ-like state	Same as above
State transfer	Same as encoding into a GHZ-like state	$t\sim \{\begin{array}{ll} r^{\alpha (\alpha -d)/(\alpha +d)} & \alpha \in (d,d+1][24]\\ r^{\alpha /(2d+1)} & \alpha \in (d+1,2d+1)[24,25] \end{array}$	Same as above
State transfer (no initialization)	$t≳\{\begin{array}{ll} r^{\left(2\alpha -2d)/(2\alpha -d+1\right)} & \alpha \in (d,2d][58]\\ r^{\alpha -2d} & \alpha \in (2d,2d+1)[26]\\ r^{\alpha -1} & \alpha \in (1,2),d=1[59]\\ r & \alpha \in (2,3),d=1[59] \end{array}$	t~r∀α∈(d,2d+1)	Not applicable
